# Validity of anthropometric equations to estimate infant fat mass at birth and in early infancy

**DOI:** 10.1186/s12887-017-0844-6

**Published:** 2017-03-27

**Authors:** Jennifer S. Cauble, Mira Dewi, Holly R. Hull

**Affiliations:** Department of Dietetics and Nutrition, School of Health Professions, University of Kansas Medical Center, 3901 Rainbow BLVD, Mail Stop 4013, Kansas City, KS 66160 USA

**Keywords:** Prediction equations, Infant, Fat mass, Anthropometrics, ADP, Skinfolds

## Abstract

**Background:**

In newborns and children, body fat estimation equations are often used at different ages than the age used to develop the equations. Limited validation studies exist for newborn body fat estimation equations at birth or later in infancy. The study purpose was to validate 4 newborn fat mass (FM) estimation equations in comparison to FM measured by air displacement plethysmography (ADP; the Pea Pod) at birth and 3 months.

**Methods:**

Ninety-five newborns (1–3 days) had their body composition measured by ADP and anthropometrics assessed by skinfolds. Sixty-three infants had repeat measures taken (3 months). FM measured by ADP was compared to FM from the skinfold estimation equations (Deierlein, Catalano, Lingwood, and Aris). Paired t-tests assessed mean differences, linear regression assessed accuracy, precision was assessed by R^2^ and standard error of the estimate (SEE), and bias was assessed by Bland-Altman plots.

**Results:**

At birth, FM measured by ADP differed from FM estimated by Deierlein, Lingwood and Aris equations, but did not differ from the Catalano equation. At 3 months, FM measured by ADP was different from all equations. At both time points, poor precision and accuracy was detected. Bias was detected in most all equations.

**Conclusions:**

Poor agreement, precision, and accuracy were found between prediction equations and the criterion at birth and 3 months.

## Background

Body weight for length measures are commonly used to identify those at risk for obesity development. Though widely used, weight for length measures do not differentiate the proportion of body weight that is fat mass (FM) or fat free mass (FFM) and are poor indicators of nutritional status and growth [1, 2]. To assess obesity risk and answer critical questions related to nutritional status and growth, infant body composition measurement is required. A number of techniques are available to measure infant body compositon; however the equipment is expensive, not widely available, impractical to use in large population studies, and often located in research laboratories. To combat these limitations, anthropometric equations have been developed and are commonly used in large epidemiologic or multi-site cohort studies [3–9].

There is a track record for studies to use equations that have been developed in one age group and then validated in another age group [10–12] or equations are used in age groups where they were not developed or validated [9, 13, 14]. Some equations have been validated whereas other equations lack proper validation data suggesting they should not be used. The Dauncey et al. [15] equation was developed in newborns and validated by Kulkarni et al. [10] in infants 6–18 months old, and used in one year old infants [16]. The Slaughter et al. equation [17] was developed in children and youth aged 8–29 years and was validated in infants from birth to four months of age [11, 18] and 6–7 year old children [19], and was used in children aged 3–5 years [13, 14]. The Goran et al. equation [19] was developed in children 4 to 10 years old and validated by Hussain et al. [12] in 14 year old children. The Westrate et al. [20] equation was developed in 7 to 10 year old children but used to estimate infant FM [4, 6, 21] even though the equation was reported to result in significant bias when applied to young infants [22]. It is important to use equations that have validation data in the age range they are being applied and have validated results otherwise incorrect conclusions may be drawn from the data.

Recently developed and commonly used infant FM estimation equations include Deierlein et al. [23], Catalano et al. [24], Lingwood et al.[11], and Aris et al. [25]. Limited validation studies have been completed in these equations. The Deierlein et al. equation has never been validated [23], while the Lingwood and Aris equations have not been validated in a population other than what was used to develop the equation [11, 25]. It is important to validate equations in the age group they were developed to verify accuracy, in age groups where equations are used but have not been validated, and in age groups where the equations may be used [26]. Therefore, the aim of this study was to validate the Deierlein et al. [23], Catalano et al. [24], Lingwood et al. [11], and Aris et al. [25] infant anthropometric equations at birth and 3 months in comparison to body fat measured using air displacement plethysmography (ADP).

## Methods

### Participants

The study analyzed data from 95 infants that participated in the Pregnancy Health Study approved by the Institutional Review Board (#13126 and 12793). For the parent study, women were recruited and followed during pregnancy. Of the 95 infants who completed the first visit (1–3 days after birth), 63 came back for their second visit at 3 months.

Ethnicity was self-selected by the mother using a questionnaire. The mother was asked to self-identify her own ethnicity and the ethnicity of the father of the baby. Next, the mother self-identified the ethnicity of her parents and the parents of the baby’s father (grandparents to the infant). The following categories were available for selection: Asian, non- Hispanic Black (African American), non-Hispanic White, and Hispanic. When all selected categories matched for parents and grandparents, the infant was identified by that category. If multiple categories were identified, the infant was classified as “other.”

### Study procedures

Women with a healthy full term (>37 weeks) pregnancy were recruited at a prenatal visit at the OB clinic. The inclusion criteria were: maternal age 18–40 years, singleton pregnancy, and body mass index (BMI) >18 kg/m^2^. Women were excluded if they had a serious pregnancy complication, developed gestational diabetes, pre-eclampia, gestational hypertension, or the infant was born with a congential anomaly known to effect fetal growth. Maternal data were collected at visits to the research laboratory in the first, second and third trimesters. Infant body composition was assessed after discharge from the maternity unit. Informed written consent was obtained before any measurement was completed. Women gave consent on behalf of their infant.

### Estimation equations

Four different infant FM estimation equations were validated at birth (1–3 days) and at 3 months. The equations were Deierlein et al. [23], Catalano et al. [24], Lingwood et al. [11], and Aris et al. [25]. The Lingwood equation yielded predicted FFM, therefore FM was calculated by subtracting the predicted FFM from body weight. The details regarding equation predictor variables, reference method used when developing the equation, and details on the sample used to develop the equation are listed in Table 1. Gender, infant age, infant ethnicity, body weight, length and various skinfolds are examples of the variables used in the equations. All equations were developed in newborns ranging in age from 0 to 4 days old. Catalano et al. used total body electrical conductivity (TOBEC) as the reference method to develop the prediction equations while all other equations used ADP as the reference method.

**Table 1:** Anthropometric equations to estimate infant fat mass (kg)

Reference	Equations	Reference method	N subjects	Subject age of range
Deierlein et al. [21]	−0.012 – 0.064*gender(1 = male; 0 = female) + 0.0024*age (days) – 0.150*body weight (kg) + 0.055*body weight^2^ (kg)^2^ + 0.046*ethnicity (1 = Hispanic; 0 = not Hispanic) + 0.020*sum of 3 skinfolds (triceps, subscapular and thigh)	ADP	128	1-3 days
Catalano et al. [20]	0.54657 + 0.39055 * Birth weight (g) + 0.0453*Flank Skinfold (mm) – 0.03237*Length (cm)	TOBEC	194	1-3 days
Lingwood et al. [11]	FFM = 0.057 + 0.646 * weight (kg) - 0.089 * gender (1 = male; 2 = female) + 0.009 * length (cm) FM = weight - FFM	ADP	77	0-4 days
Aris et al. [22]	−0.022 + 0.307 * weight (kg) - 0.077 * gender (1 = male; 0 = female) - 0.019 * gestational age (week) + 0.028 * subscapular skinfold (mm)	ADP	88	1-3 days

*indicates multiplication in the scientific equation

### Anthropometric measures

All measurements were collected using standardized procedures to our Laboratory [27] and took place on the same day as the body composition assessment by ADP. All skinfolds were identified by anatomical landmarks and taken on the right side of the body using Lange calipers (Beta Technology, Santa Cruz, CA). Skinfolds were taken in order from head to toe and then repeated in that same order. If two skinfold measurements differed by more than 0.5 mm, a third measurement was repeated. The two measurements within 0.5 mm were averaged and used for the analysis. Biceps and triceps skinfolds were measured at the midline of the anterior and posterior surface of the arm, respectively, on the mid-point between acromial process of the scapula and olecranon process of the ulna. The subscapular skinfold was measured at the lower angle of the scapula. The thigh skinfold was measured at the mid-point between the proximal edge of the patella (knee cap) and inguinal crease at the anterior surface of the thigh. The flank skinfold was measured immediately above the iliac crest at the mid-axillary line. Technicians completed annual anthropometric training and validity statistics were calculated. Three testers were responsible for collecting the anthropometric data. Intraclass correlations (ICC) were calculated for each skinfold site. The range of ICC’s for the skinfold sites ranged from 0.83 to 0.96 and the technical error of measurement ranged from 0.23 to 0.34.

### Air displacement plethysmography

Air displacement plethysmography (Pea Pod®, Software version 3.5.0, 2015, CosMed, Concord, CA) was used to measure infant body composition. Body weight was measured using the integrated scale and measured to the nearest 0.01 kg. Body composition was determined by measuring body volume and calculating body density. A cap was worn to minimize air trapped in the hair. All clothing and the diaper were removed before the body volume measurement. After the infant body volume was acquired, body density was calculated and converted to percentage body fat (%fat) using gender specific equations by Fomon et al. [28]. Air displacement plethysmography is a valid technique to assess infant body composition and was validated against the gold standard 4 compartment model (4C) [29] and against total body water using deuterium dilution [30]. No differences were found for percentage body fat when compared to the 4C model or total body water [29, 30]. Infant ages in these validation studies ranged from 0.4 to 23 weeks which includes the ages in our sample. Therefore, ADP will be considered the criterion measure in this validation study.

### Statistical analyses

Means and standard deviations were calculated for all continuous variables. For these analyses, ADP was considered the criterion. Differences between the criterion measure and each estimation equation were assessed using paired t-tests. Regression analysis was used to assess the accuracy between the criterion and each of the equations. To be considered accurate, the regression line relating the two measurements should have a slope equal to 1.0. A slope that deviates significantly from 1.0 suggests a unit change in the estimation equation does not correspond to a unit change in the criterion. Precision was assessed by R^2^ and standard error of the estimate (SEE). A SEE between 2 and 3% of fat mass is desirable and classified as very good whereas a SEE >4.0% is considered poor [31]. In our sample, an SEE value of 3% is 0.015 kg at birth and 0.067 kg at 3 months. An R^2^ value should exceed 0.64. A R^2^ value <0.64 suggests poor agreement between the two methods and poor predictive value of the equations relative to the criterion. Bland Altman was used to assess agreement between the methods [32]. This analysis involves an assessment of the correlation or the measure of strength for the relationship between the mean of the criterion and each equation (mean infant FM criterion + mean infant FM from each equation/2) correlated to the difference between the equation estimated infant FM and the criterion measured FM. A non-significant correlation suggests no bias in the technique across the range of fatness. This provides insight into how much the equation estimated FM differs and relates to the criterion measured FM. Statistical analyses were conducted using SPSS version 20.0 (IBM, Armonk, NY). Statistical significance was set as *p* ≤ 0.05.

## Results

Sample descriptive statistics are reported in Table 2. Fat mass assessed by the criterion and calculated for all equations are presented in Table 3. The average FM measured by the criterion was 0.374 kg at birth and 1.664 kg at visit 2.

**Table 2:** Maternal and infant descriptive statistics for the sample at birth and at 3 months

	Birth (*n* = 95)	3 months (*n* = 63)
Maternal age (years)	28.9 ± 4.8	29.9 ± 4.0
Maternal pre-pregnancy BMI (kg/m^2^)	25.8 ± 6.1	25.4 ± 5.6
Maternal gestational weight gain (kg)	15.6 ± 6.0	15.2 ± 5.5
Gestational age (wks)	39.24 ± 2.8	39.20 ± 3.3
Birthweight (g)	3497.5 ± 404.6	3539.0 ± 445.2
Birth length (cm)	50.4 ± 2.1	50.0 ± 4.8
Male (%)	42 (44.2%)	26 (41.3%)
Infant age (days)	2.3 ± 1.3	117.2 ± 23.2
Ethnicity		
White	68 (71.6)	51 (81.0)
African-American	14 (14.7)	5 (7.9)
Hispanic	8 (8.4)	4 (6.3)
Asian	5 (5.3)	3 (4.8)
Body weight at assessment (g)	3272 ± 388.2	6595.1 ± 841.4
Length at assessment (cm)	50.4 ± 2.1	63.9 ± 4.4
Skinfolds		
Triceps (mm)	5.6 ± 1.5	11.1 ± 2.9
Biceps (mm)	4.4 ± 1.2	7.4 ± 2.1
Subscapular (mm)	5.3 ± 1.4	7.9 ± 2.0
Thigh (mm)	7.7 ± 1.9	20.2 ± 4.4
Percentage body fat by criterion (%fat)	11.2 ± 4.3	25.0 ± 5.1
Fat mass by criterion (kg)	0.374 ± 0.17	1.664 ± 0.44
Fat-free mass by criterion (kg)	2.897 ± 0.29	4.93 ± 0.63
Values are mean ± SD.

**Table 3:** Fat mass assessed by the different methods at birth and 3 months

Method	Fat mass (kg)	
	Birth (*n* = 95)	3 months (*n* = 63)
Criterion	0.374 ± 0.171	1.664 ± 0.433
Deierlein	0.488 ± 0.154^*^	4.989 ± 0.987^*^
Catalano	0.362 ± 0.138	1.392 ± 0.301^*^
Lingwood	0.330 ± 0.137^*^	1.378 ± 0.261^*^
Aris	0.340 ± 0.157^*^	1.433 ± 0.276^*^

Values are mean ± SD. Criterion method was ADP

* Significant difference from the criterion method *p* < 0.05

### Fat mass measured at birth

At birth, mean differences were found between all equations (*p* < 0.05) and the criterion except for the Catalano equation (Table 3). Table 4 reports results for accuracy and precision assessed by regression. The slope measured by regression from all equations against the criterion differed from 1 (*p* < 0.0001). Poor agreement and precision was found between methods with low R^2^ values ranging from 0.55 to 0.63 and high SEE values ranging from 0.106 to 0.116 kg.

**Table 4:** Results for regression and Bland Altman analysis for comparison of the criterion method and the fat mass estimation equations at birth and 3 months

Comparison	Regression analysis			Bland and Altman				
	Slope	R^2^	*p*-value	SEE	Mean bias ± SD	95% limits of agreement	Pearson Correlation (r)	*p*-value*
Birth (*n* = 95)								
Deierlein vs Criterion	−0.87	0.61	<0.0001	0.108	0.114 ± 0.109	−0.010 - 0.328	−0.17	0.099
Catalano vs Criterion	0.92	0.55	<0.0001	0.116	−0.012 ± 0.116	−0.240 - 0.215	−0.31	0.002
Lingwood vs Criterion	0.93	0.55	<0.0001	0.116	−0.045 ± 0.116	−0.272 - 0.183	−0.33	0.001
Aris vs Criterion	0.87	0.62	<0.0001	0.106	−0.034 ± 0.107	−0.245 - 0.176	−0.15	0.140
3 months (*n* = 63)								
Deierlein vs Criterion	0.29	0.42	<0.0001	0.333	3.325 ± 0.784	1.789 - 4.862	0.77	<0.0001
Catalano vs Criterion	1.02	0.50	<0.0001	0.308	−0.271 ± 0.306	−0.871 - 0.328	−0.47	<0.0001
Lingwood vs Criterion	1.24	0.55	<0.0001	0.294	−0.286 ± 0.298	−0.871 - 0.299	−0.63	<0.0001
Aris vs Criterion	1.15	0.52	<0.0001	0.303	−0.230 ± 0.303	−0.824 - 0.363	−0.57	<0.0001

* Significance for the correlation of the strength for the relationship between the mean of the criterion and each equation correlated to the difference between the equation estimated infant fat mass and the criterion measured fat mass. A non-significant correlation suggests no bias in the technique across the range of fatness

**Fig. 1:**
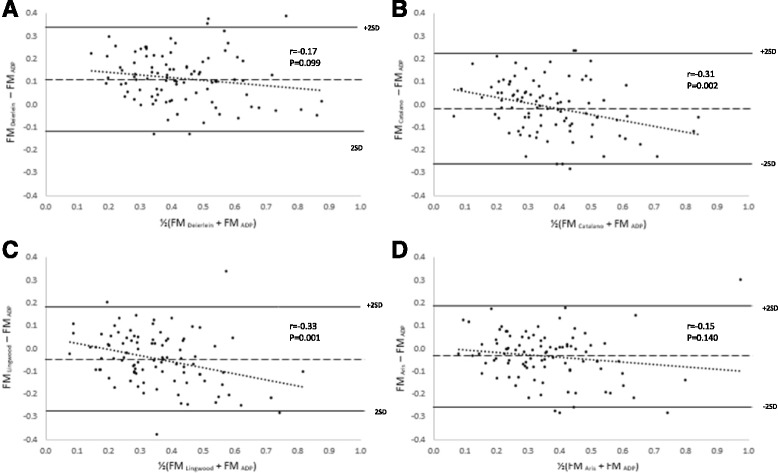
Bland-Altman plot of the absolute weight of FM (kg) estimated by the prediction equations from Deierlein et al. (**a**), Catalano et al. (**b**), Lingwood et al. (**c**) and Aris et al. (**d**) against the criterion at birth. The *middle dashed line* represents the mean difference between the infant prediction equations and the criterion. The *upper* and *lower solid line* represents ±2SD from the mean

A residual plot analysis of the predictive error (Bland-Altman plot) from the equations against the criterion is reported in Table 4 and Fig. 1. Bias was detected for the Catalano and Lingwood equations. This suggested the Catalano and Lingwood equations ovestimated FM at lower FM values and underestimated FM values at greater FM values. For the Deierlein equation, no bias was detected though the value approached significance (*p* = 0.099). No bias was detected for the Aris equation (*p* = 0.140). Even though no bias was detected in the Aris equation, the 95% limit of agreement was wide.

### Fat mass measured at 3 month

At 3 months, the mean FM for all equations differed from the criterion (*p* < 0.0001) (Table 3). Table 4 reports results for accuracy and precision assessed by regression. The slope measured by regression from all equations against the criterion differed from 1 (*p* < 0.0001). At 3 months, R^2^ values were poor ranging from 0.43 to 0.55 and the SEE values were high ranging from 0.095 to 0.303 kg.

**Fig. 2:**
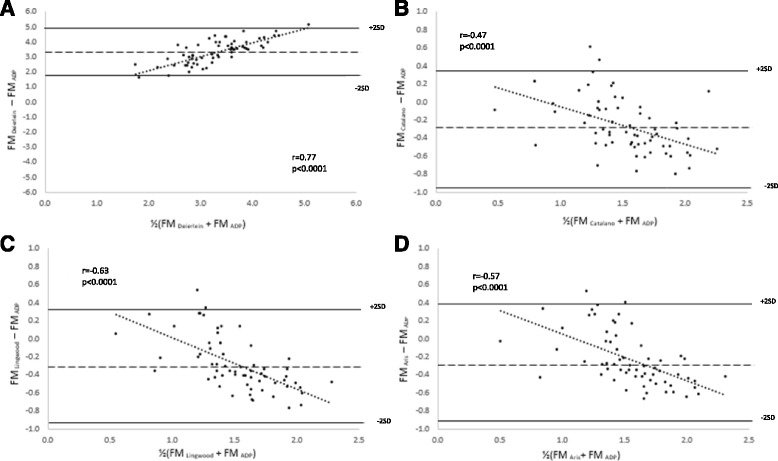
Bland-Altman plot of the absolute weight of FM (kg) estimated by the prediction equations from Deierlein et al. (**a**), Catalano et al. (**b**), Lingwood et al. (**c**) and Aris et al. (**d**) against the criterion at 3 months. The *middle dashed line* represents the mean difference between the infant prediction equations and the criterion. The *upper* and *lower solid line* represents ± 2SD from the mean

Bias was detected for all equations when compared to the criterion (*p* < 0.0001; Table 4 and Fig. 2). The data suggests that the Deierlein equation overestimates FM at all values with the estimations being greater at higher FM values. Conversely, the Catalano, Lingwood, and Aris equations overestimate FM at lower FM values and underestimates FM at higher FM values.

## Discussion

This study validated equations to predict infant FM against a validated technique (ADP) in newborns at birth (1 to 3 days) and in infants at 3 months. The equations we chose to validate were all developed in infants aged 0–4 days old [11, 23–25]. We sought to test the validity of the equations not only at birth but also at a later age range (3 months) since research studies have used the Catalano et al. equation at birth [24] and at 4–6 weeks old [9]. Further, it is not uncommon to use prediction equations in other age populations than they were developed.

At birth, mean differences were detected between the criterion and all equations except for Catalano et al. At 3 months, mean differences were detected between the criterion and all equations. The predictive error (SEE) was poor for all equations at birth. The predictive error increased just 3 months later. Poor agreement was found between all equations at birth and 3 months as represented by R^2^ values <0.64. At birth, bias was detected for the Catalano and Lingwood equations while at the 3 month visit, bias was detected in all equations.

We wanted to compare the results of our study to other studies that have validated the infant prediction equations we tested. Catalano et al. [5] recently validated the Catalano et al. equation [24] against ADP in sample of newborns not used to develop the equation. They reported a better correlation than was found in this study (R^2^ = 0.69 vs 0.55). To our knowledge, the Deierlein equation has never been validated and the Aris and Lingwood equations have only been cross validated using a portion of the sample that was used to develop the equations. Since there are limited data on the equations we validated, we identified the Dauncey et al. equation [15] as another commonly used infant prediction equation to compare precision and accuracy results. We could not validate the Dauncey et al. equation [15] due to missing variables in our dataset. Validation studies [24, 33, 34] found a similar range of poor agreement (R^2^ = 0.40 – 0.61) as was found in our study (R^2^ = 0.55 – 0.63). Even though poor agreement was found the equation is used in research studies [35, 36].

We wanted to explore potential reasons why mean differences and poor agreement and bias have been detected in infant prediction equations. The period of early infancy presents a period of rapid infant growth with a wide range of inter-individual variability. A range of infant growth exists due to gender differences and the infant feeding method (formula vs. breastfeeding) [37]. Differences are especially apparent early from 1–4 months of age where formula fed infants gain more body weight when compared to breast fed infants [38, 39]. Though the growth rate of breast fed infants is slower when compared to formula fed infants, breast fed infants have greater FM from birth to 9 months when compared to formula fed infants [40]. In addition, Shepherd et al. [39] found gender differences in body composition changes in formula fed infants. In the early months, the extra body weight gained in males was detected as FM whereas in females they gained FFM. None of the infant prediction equations validated included feeding method as a predictor variable, likely because they were developed in infants 0–4 days old.

The feeding method may also cause error in assessing infant body composition at birth. Macdonald et al. [41] assessed differences in body weight loss and recovery of birth weight in formula fed and breast fed newborns. The timing of loss was the same between the two groups, but breast fed infants lost 6.6% of their birth weight at day 3 whereas formula fed infants only lost 3.5% of their body weight at day 3. Further, regain to birth weight was slower in breast fed infants (8.3 days) compared to formula fed infants (6.5 days). It is unknown how these differences would impact the prediction of infant body composition but it is plausible the infant feeding method could create error in the prediction of infant body composition at birth.

Further support that rapid infant growth is influencing the accuracy and precision of infant prediction equations can be found in the better performance of prediction equations in children, adolescents, and teenagers. The Slaughter et al. [17] equation is commonly used to predict FM in 8 to 29 year olds. Validation studies in 5 to 19 year olds [12, 23, 42] foundgood agreement and precision (R^2^ = 0.76-0.81; SEE: 3.73%). As weight gain and body composition becomes more stable in childhood when compared to early infancy, we speculate that prediction equations are more accurate.

Another potential reason poor agreement and bias were detected may be differences in race/ethnicity between the populations. It is well published that there are differences in body composition based on race/ethnicity detected at birth through adulthood [8, 43–46]. If there is a mismatch between the validation population and the population the equations are being applied, errors in estimation may occur. Only the Deirerlein equation included ethnicity as a predictor variable [23]. The race/ethnic breakdown of the Deirerlein pouplation was ~42% Caucasian, 6% African American, 20% Hispanic, 10% Asian, and 22% other. The populations used to develop the other equations were the following: Catalano was primarily Caucasian (64%) and data were collected in the United States [24], the Lingwood equation was developed from an Australian sample that was 89% Caucasian [11], and the Aris sample was an Asian population from Singapore that was comprised of Chinese, Malay, and Indian newborns. Our sample was primarily Caucasian (~71%) and collected in the United States. The Catalano equation included a population similar to ours, however, the other equations were developed with populations that had race/ethnic or location differences. This may contribute to the poor performance of the equations in our sample.

### Strength and limitation

One strength of our study is that we compared the estimation equations to ADP, which is a technique that was developed and validated [29, 30] specifically to assess infant body composition. Additionally, our analysis validated and discussed multiple equations that are used to assess infant body composition. A third strength is that we sought to validate this equation at multiple infant ages. Fat mass measurements are needed across infancy and this comparison provides insight into accuracy at time points other than at birth. A potential limitation to our study was the inability to assess the validity of the Dauncey et al. [15] equation. This equation is commonly used and providing a comparison within the context of the other infant prediction equations would have been valuable.

## Conclusions

In conclusion, differences were found between the prediction equations and criterion and poor accuracy, agreement and precision was detected. Equipment to measure infant body composition is not widely available and therefore estimation equations are commonly used. The equations we validated performed poorly therefore caution should be used when interpreting data collected with these equations to avoid erroneous conclusions. Development of equations that provide more accurate estimates is desperately needed.

## Abbreviations

%fat: Percentage body fat
4C: 4 compartment model
ADP: Air displacement plethysmography
FFM: Fat free mass
FM: Fat mass
SEE: Standard error of the estimate
