# Correction to: Validity of anthropometric equations to estimate infant fat mass at birth and in early infancy

**DOI:** 10.1186/s12887-020-1989-2

**Published:** 2020-02-28

**Authors:** Jennifer S. Cauble, Mira Dewi, Holly R. Hull

**Affiliations:** Department of Dietetics and Nutrition, School of Health Professions, University of Kansas Medical Center, 3901 Rainbow BLVD, Mail Stop 4013, Kansas City, KS 66160 USA

**Correction to: BMC Pediatr**

**https://doi.org/10.1186/s12887-017-0844-6**

Following the publication of article by Cauble et al. [1], typographical errors were discovered. The Lingwood et al. and Deierlein et al. equations were listed incorrectly. To avoid confusion to the readers, the authors propose to publish a correction. The errors and the corresponding corrections are shown below:

**The paper lists the Deierlein equation as:**

$$ -0.012-0.064\ast \mathrm{gender}\left(1=\mathrm{male};0=\mathrm{female}\right)+\mathbf{0.0024}\ast \mathrm{age}\ \left(\mathrm{days}\right)-0.150\ast \mathrm{body}\ \mathrm{weight}\ \left(\mathrm{kg}\right)+0.055\ast {\mathrm{body}\ \mathrm{weight}}^2\ {\left(\mathrm{kg}\right)}^2+0.046\ast \mathrm{ethnicity}\ \left(1=\mathrm{Hispanic};0=\mathrm{not}\ \mathrm{Hispanic}\right)+0.020\ast \mathrm{sum}\ \mathrm{of}\ 3\ \mathrm{skinfolds}\ \left(\mathrm{triceps},\mathrm{subscapular},\mathrm{and}\ \mathrm{thigh}\right) $$

**The correct Deierlein equation is:**

$$ -0.012-0.064\ast \mathrm{gender}\left(1=\mathrm{male};0=\mathrm{female}\right)+\mathbf{0.024}\ast \mathrm{age}\ \left(\mathrm{days}\right)-0.150\ast \mathrm{body}\ \mathrm{weight}\ \left(\mathrm{kg}\right)+0.055\ast {\mathrm{body}\ \mathrm{weight}}^2\ {\left(\mathrm{kg}\right)}^2+0.046\ast \mathrm{ethnicity}\ \left(1=\mathrm{Hispanic};0=\mathrm{not}\ \mathrm{Hispanic}\right)+0.020\ast \mathrm{sum}\ \mathrm{of}\ 3\ \mathrm{skinfolds}\ \left(\mathrm{triceps},\mathrm{subscapular},\mathrm{and}\ \mathrm{thigh}\right) $$

**The paper lists the Lingwood equation as:**

$$ \mathrm{FFM}=\mathbf{0.057}+0.646\ast \mathrm{weight}\ \left(\mathrm{kg}\right)-0.089\ast \mathrm{gender}\ \left(1=\mathrm{male};2=\mathrm{female}\right)+0.009\ast \mathrm{length}\ \left(\mathrm{cm}\right) $$

$$ \mathrm{FM}=\mathrm{weight}-\mathrm{FFM} $$

**The correct Lingwood equation is:**

$$ \mathrm{FFM}=\mathbf{0.507}+0.646\ast \mathrm{weight}\ \left(\mathrm{kg}\right)-0.089\ast \mathrm{gender}\ \left(1=\mathrm{male};2=\mathrm{female}\right)+0.009\ast \mathrm{length}\ \left(\mathrm{cm}\right) $$

$$ \mathrm{FM}=\mathrm{weight}-\mathrm{FFM} $$
